# Chronic Obstructive Pulmonary Disease (COPD) Exacerbation With Subsequent Pneumothorax and Aortic Dissection: A Case Report

**DOI:** 10.7759/cureus.85525

**Published:** 2025-06-07

**Authors:** Rachelle Beste, Mason Bonner, Kara Bragg, Jeremy Collado

**Affiliations:** 1 Emergency Medicine, Mayo Clinic, Jacksonville, USA

**Keywords:** aortic dissection, case report, copd, pneumothorax, respiratory distress

## Abstract

Chronic obstructive pulmonary disease (COPD), pneumothorax, and aortic dissection are all potentially life-threatening intrathoracic pathologies known for presenting with a chief complaint of shortness of breath. The following case involves a 74-year-old male who presented to the emergency department with acute dyspnea. He was found to have concurrent COPD exacerbation, pneumothorax, and aortic dissection. While pneumothorax is a known complication in older patients with underlying COPD, aortic dissection is an exceedingly rare complication. Healthcare providers should consider the possibility of concurrent multiple disease processes in the workup of acute shortness of breath.

## Introduction

The following case involves a 74-year-old man who presented to the emergency department in acute respiratory distress. He was found to have a combination of COPD exacerbation, pneumothorax, and aortic dissection. While pneumothorax is a known complication in older patients with underlying COPD [1], aortic dissection is an exceedingly rare complication of pneumothorax [2]. This multifactorial presentation of shortness of breath, during which COPD exacerbation may have served as a catalyst for concurrent pneumothorax, and aortic dissection highlights the need for caution and a broad differential diagnosis in the evaluation of acute dyspnea. It further serves to highlight the possibility of multiple disease processes presenting concurrently.

## Case presentation

A 74-year-old man with a history of COPD, cigarette smoking, cirrhosis, hypothyroidism, and nonmelanoma skin cancers presented to the emergency department for shortness of breath. He had been diagnosed with pneumonia approximately two weeks prior and had completed a five-day course of doxycycline with symptom improvement. He had not been prescribed corticosteroids.

On the evening of his presentation, he awoke with a coughing fit and worsening shortness of breath. When emergency medical services arrived at his home, the patient was tripoding and had oxygen saturations between 75% and 80% on room air. He reported some associated chest tightness with a nonproductive cough but denied further fevers after initiating antibiotic therapy. He denied prothrombotic risk factors. He was placed on a non-rebreather and treated with magnesium 2 g, methylprednisolone 125 mg, and nebulized albuterol 5 mg.

**Figure 1:**
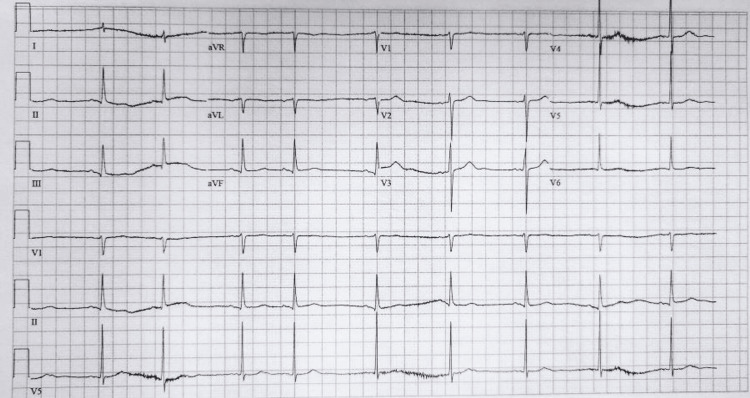
Sinus bradycardia with sinus arrhythmia, a rate of 58, and nonspecific T-wave changes without signs of acute ischemia

Upon arrival at the ED, the patient was immediately started on noninvasive positive pressure ventilation (NIPPV) for his difficulty breathing. Vital signs included a blood pressure of 123/73, pulse rate of 59, afebrile with a respiratory rate of 30, and oxygen saturation of 92%. His physical examination was notable for moderate respiratory distress, tachypnea, scattered wheezes, and diminished lung sounds on the right. Electrocardiography showed sinus bradycardia with sinus arrhythmia, a rate of 58, and nonspecific T-wave changes without signs of acute ischemia (Figure 1).

**Figure 2:**
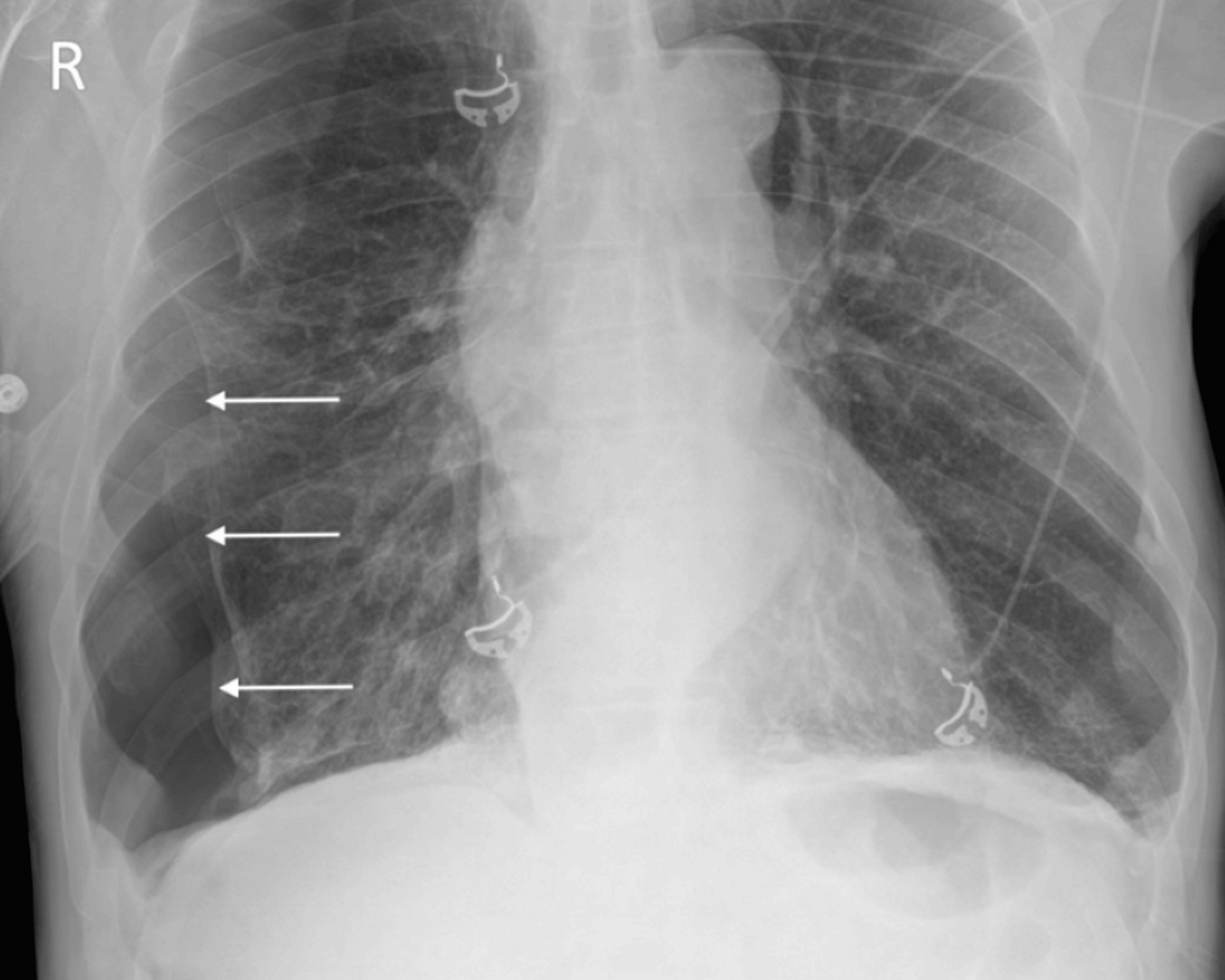
Moderate, right-sided pneumothorax without mediastinal shift

Portable chest radiography showed a moderate, right-sided pneumothorax without mediastinal shift (Figure 2). NIPPV was discontinued, and he was placed on a non-rebreather mask at 10L.

**Figure 3:**
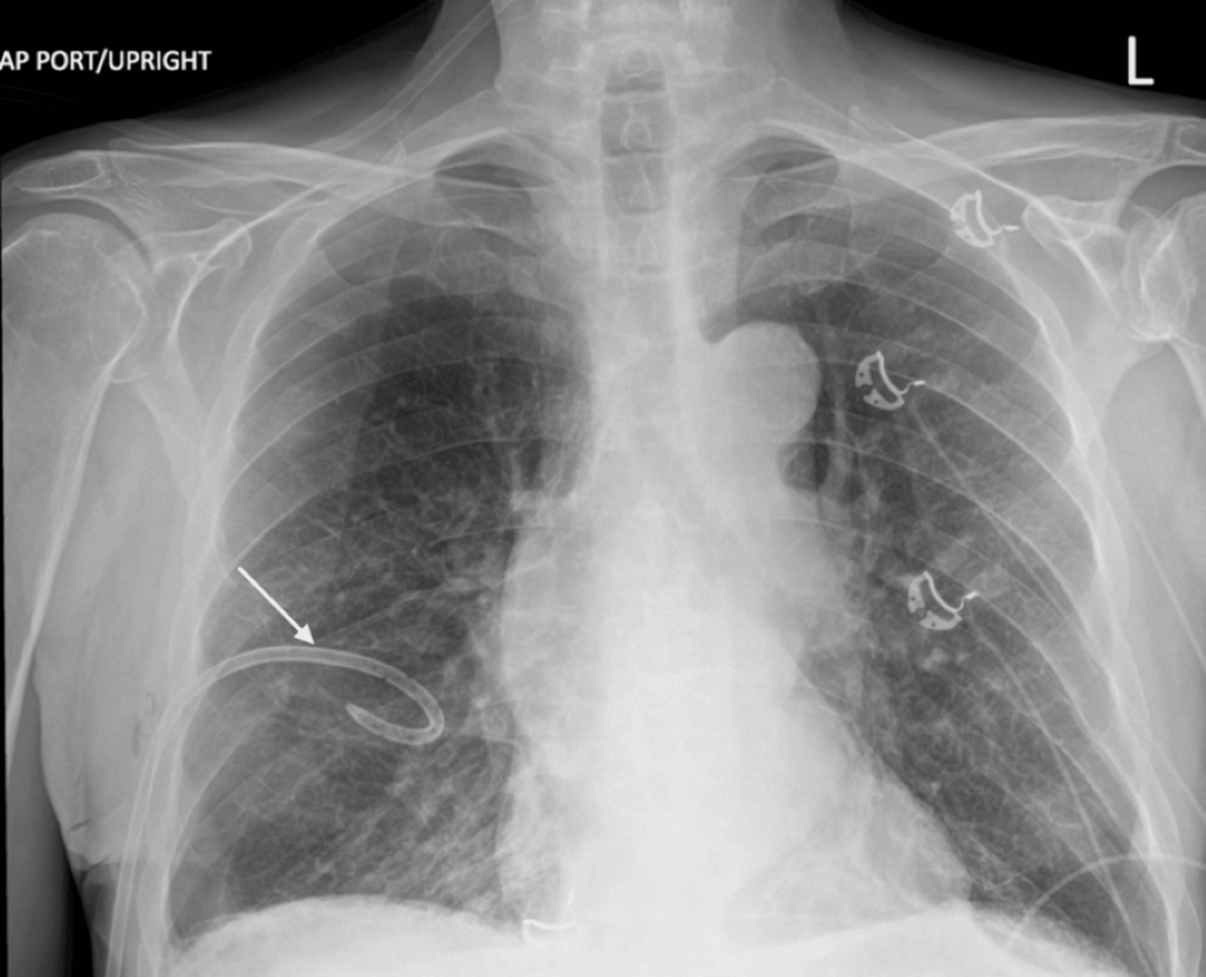
Chest X-ray showing near-complete resolution of pneumothorax after chest tube placement, with underlying emphysematous changes

A 7 French percutaneous small-bore chest tube was placed without difficulty. He immediately showed signs of clinical improvement following chest tube placement and was transitioned to a nasal cannula. Repeat radiography showed near-complete resolution of his pneumothorax with underlying emphysematous changes (Figure 3).

Laboratory results were notable for a mildly elevated initial troponin T of 42 ng/L and an elevated D-dimer of greater than 42,000 ng/mL (Table 1). Arterial blood gas (ABG) prior to chest tube insertion was reassuring with a pH of 7.39, pCO2 44, pO2 307, and bicarbonate of 26 on bilevel positive airway pressure (BiPAP) with a fraction of inspired oxygen (FiO2) of 100%.

**Table 1:** D-dimer and troponin-T values and reference ranges

LAB	Result	Reference Range
D-dimer	42,000 ng/L	<=500 ng/L
Troponin T	22 ng/L	<=15 ng/L

**Figure 4:**
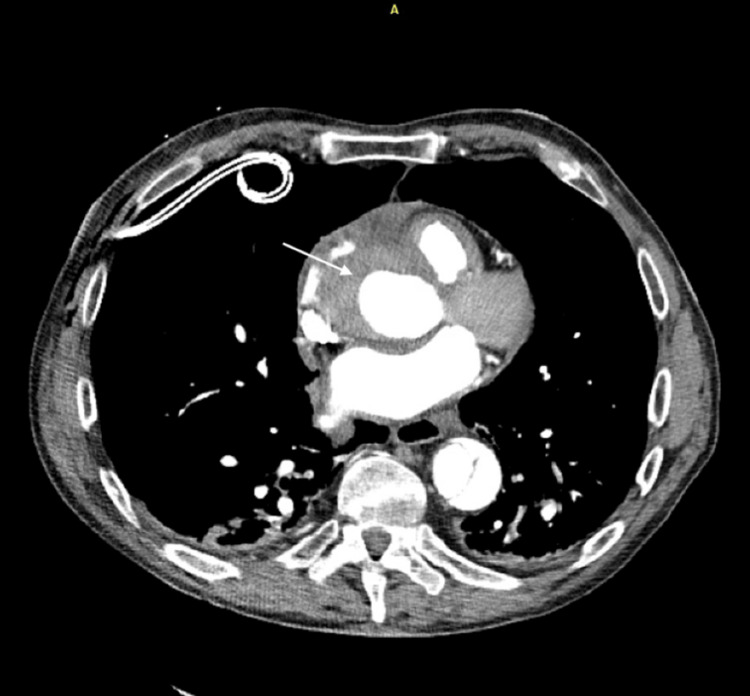
CTA showing type B dissection originating just distal to the left subclavian artery and extending to the infrarenal aorta, a retrograde intramural hematoma involving the ascending aorta and a small pericardial effusion A: anterior

Computed tomography angiography (CTA) of the pulmonary arteries showed no pulmonary embolism but did reveal a Stanford type B aortic dissection (Figure 4). Cardiothoracic and vascular surgery teams were consulted, and the patient underwent a repeat CTA dissection protocol to determine the extent of the dissection flap. Imaging showed a complex type B dissection originating just distal to the left subclavian artery and extending to the infrarenal aorta, a retrograde intramural hematoma involving the ascending aorta, and a small pericardial effusion.

**Figure 5:**
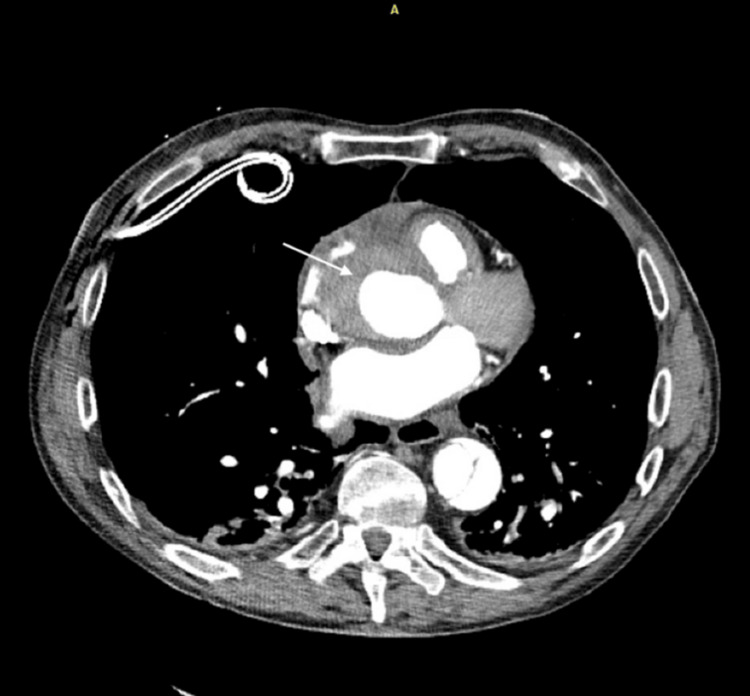
Computed tomography angiography showing a complex aortic dissection with retrograde intramural hematoma (arrow) involving the ascending aorta A: anterior

The patient was transferred to the intensive care unit for close hemodynamic monitoring while awaiting a decision regarding potential surgical management. He and his family elected to continue medical support with repeat imaging to determine flap stability, as he was asymptomatic with no evidence of malperfusion. Repeat CTA showed a stable proximal hematoma with a slight extension of type B flap dissection (Figure 5). Given these findings, the patient and his family decided to proceed with surgery. He underwent a median sternotomy with direct right axillary cannulation, ascending aortic dissection repair, aortic valve resuspension, aortic debranching, and direct anastomosis of the innominate and common carotid arteries approximately 24 hours after initial diagnosis. The procedure was notable for severe bleeding and hypotensive episodes. He received prothrombin complex concentrate in addition to multiple blood products, methylene blue, and vasopressors. Given hemodynamic instability and concern for hemorrhage, he underwent a second procedure the following day for exploration, hematoma evacuation, and control of bleeding. Unfortunately, his postoperative course continued to be complicated by bleeding, refractory hemodynamic instability, sepsis, and multiorgan system failure. He remained on mechanical ventilation, vasopressor support, and continuous renal replacement therapy with no clinical improvement despite aggressive measures. He was transitioned to comfort care by his wife on hospital day 15 and died shortly thereafter.

## Discussion

Shortness of breath, among respiratory concerns in general, is a common chief complaint in the emergency department [3]. The etiology of shortness of breath is often multifactorial, and differential diagnosis considerations carry substantial associated morbidity and mortality [4]. Generally, clinical presentation, physical examination, and recent history guide diagnosis. In this case, our patient presented in hypoxic respiratory distress with a history of COPD and had recently been treated for pneumonia. His physical examination initially suggested COPD exacerbation and concomitant pneumothorax, given wheezing and decreased right-sided breath sounds. With data suggesting multiple etiologies for his symptoms, the differential diagnosis was broad, and a multisystem approach was taken in his medical evaluation.

A literature review yielded three case reports detailing the concurrence of pneumothorax and aortic dissection [2,5,6]. One of the proposed physiologic mechanisms involves aortic dissection resulting from increased intrathoracic pressures in the setting of pneumothorax. In all three case reports, the patients had a prolonged history of smoking (>20 years) or were documented to smoke at least one pack per day. In addition, two of the case reports documented the presence of bullae, a common complication of prolonged smoking known to be associated with pneumothorax [7]. There are case reports of aortic dissection secondary to persistent coughing associated with COPD, leading to support for linking these two phenomena [8].

It is significant to note that the patient did not present with acute chest pain, one of the leading symptoms associated with aortic dissection [9]. There are reports of aortic dissection cases with shortness of breath in the absence of chest pain. This reinforces the need to expand the differential to include dissection even in the presence of other diagnoses that may account for the shortness of breath such as pneumothorax in this case [9].

The significance of this case is the simultaneous presentation of COPD, pneumothorax, and aortic dissection. There are reports of persistent cough secondary to pneumonia/COPD exacerbation leading to aortic dissection [8]. Our patient described COPD exacerbation symptoms with a severe coughing fit prior to acute decompensation. This coughing exacerbation could account for the increase in intrathoracic pressure from a pneumothorax, secondary to ruptured bullae, and may have directly led to an aortic dissection [2,8].

At present, this is the fourth known report of a combined pneumothorax and aortic dissection in the literature. However, this is the first case of a patient having concurrent COPD exacerbation, pneumothorax, and aortic dissection. Our case highlights that COPD can be associated with both serious complications: pneumothorax and aortic dissection. This cluster of comorbidities should be on the differential in patients presenting with shortness of breath, even in the absence of chest pain [7,10].

## Conclusions

Healthcare providers should be cautious in the management of acute respiratory distress, as multiple etiologies can present concurrently, especially in the setting of COPD and its associated preexisting conditions and possible sequelae. A broad differential diagnosis should be considered. COPD is commonly associated with pneumothorax as a complication, but aortic dissection is often not on the differential. This case demonstrates an example of COPD with aortic dissection, pneumothorax, and concurrent aortic dissection. While Occam’s razor is often taught as a guiding principle in medicine, we must not forget that sometimes Hickam’s dictum prevails.
